# Cajaninstilbene Acid Ameliorates Acetaminophen-Induced Liver Injury Through Enhancing Sestrin2/AMPK-Mediated Mitochondrial Quality Control

**DOI:** 10.3389/fphar.2022.824138

**Published:** 2022-03-08

**Authors:** Mingzhu Yan, Suwei Jin, Yongguang Liu, Lisha Wang, Zhi Wang, Tianji Xia, Qi Chang

**Affiliations:** ^1^ Institute of Medicinal Plant Development, Chinese Academy of Medical Sciences and Peking Union Medical College, Beijing, China; ^2^ Department of Neuroscience Care and Society, Division of Neurogeriatrics, Karolinska Institutet, Stockholm, Sweden

**Keywords:** acetaminophen, liver injury, cajaninstilbene acid, mitochondrial quality control, Sestrin2, AMPK

## Abstract

Acetaminophen (APAP)-induced liver injury (AILI) is the main cause of acute liver failure in the developed countries. The present study aimed to evaluate the therapeutic efficacy of cajaninstilbene acid (CSA), a major stilbene compound derived from the leaves of pigeon pea [*Cajanus cajan* (L.) Millsp.], against AILI. CSA (50, 75 mg/kg, p. o.) was administered to male C57BL/6 N mice 0.5 h after a toxic dose of APAP (300 mg/kg, i. p.). The direct effect of CSA on hepatocytes was tested on primary mouse hepatocytes. Serum transaminases, hematoxylin and eosin staining, TUNEL and propidium iodide staining were used to assess hepatic damage and cell death. The results demonstrated that APAP-induced liver injury was ameliorated by CSA, as evidenced by decreased alanine aminotransferase and aspartate aminotransferase levels in the serum, and fewer necrotic and apoptotic hepatocytes *in vitro* and *in vivo*. Consequently, the inflammation in response to APAP overdose was inhibited by CSA. Without affecting APAP metabolic activation, CSA interrupted the sustained JNK-Sab-ROS activation loop and alleviated oxidative stress. Additionally, CSA promoted mitochondrial quality control, including mitochondrial biogenesis and mitophagy, as revealed by increased PGC-1α, TFAM, LC3-Ⅱ, PINK1 and mitochondrial Parkin expression and decreased p62 expression. Further mechanistic investigations showed that independent of CAMKK2, LKB1-mediated AMPK activation, which was promoted by Sestrin2, might be responsible for the protective effect of CSA. Our study demonstrates that CSA alleviates APAP-induced oxidative stress and enhanced mitochondrial quality control through Sestrin2/AMPK activation, thereby protecting against AILI,.

## Introduction

Acetaminophen (N-acetyl-p-aminophenol, APAP, or paracetamol) is one of the most commonly used over-the-counter medications for pain relief and fever reduction. Given the current spread of the Coronavirus Infectious disease (COVID-19) across the world, many patients are advised to take antipyretics containing APAP to relieve headache and fever in clinical practice (Bertolini et al., 2020). It is very safe when taken as directed, but overdose can lead to hepatotoxicity and even acute liver failure (ALF) (Larson, 2007). According to the FDA, APAP-induced liver injury (AILI) is today the leading cause of acute liver failure in America, as well as the other developed countries (Bernal and Wendon, 2013). It is estimated that one hundred thousand calls to Poison Control Centers resulted from AILI per year, and among which fifty thousand patients are admitted to emergency room in the United States (Lee, 2017).

When taken at therapeutic doses, APAP is mainly esterified in the liver to form glucuronides and/or sulfates (Prescott, 1980). Once the capacity for esterification is saturated after APAP overdose, APAP metabolism switched to the phase Ⅰ metabolic pathway, resulting in formation of a highly reactive and toxic intermediate metabolite N-acetyl-p-benzoquinone imine (NAPQI) predominantly via Cytochrome P450 2E1 (CYP2E1) (Hinson et al., 2004). NAPQI can be detoxified rapidly via glutathione (GSH) to generate a harmless product mercapturic acid, which is water soluble and can be readily excreted in the urine (Mitchell et al., 1974; Lee, 2017). However, when GSH is depleted, NAPQI binds directly to cysteine residues on cellular proteins, especially mitochondrial proteins, leading to mitochondrial dysfunction and oxidative stress, disruption of cellular integrity and ultimately cell death (Jollow et al., 1973; Yan et al., 2018). At present, N-acetylcysteine (NAC), a GSH precursor, is the only approved antidote by FDA for treating AILI. Unfortunately, the curative effect of NAC decreases markedly after the metabolic activation of APAP. Moreover, the side effect limits its clinical application (Yoon et al., 2016). Thus, searching for new therapeutics, especially new drugs targeting mitochondria, is critically needed.

Cajaninstilbene acid (CSA) is a natural stilbene compound isolated from the leaves of pigeon pea [*Cajanus cajan* (L.) Millsp.]. The pigeon pea is commonly used in animal husbandry and food industry, its leaves is traditionally used as medicine to treat trauma, burn wounds and bedsore. Hence, it is not surprising that CSA, as a major bioactive component in pigeon pea leaves, exhibits various biological activities including anti-inflammatory (Huang et al., 2016), anti-osteoporosis (Sun et al., 2019), anti-tumor (Fu et al., 2015), anti-bacterial (Lu et al., 2020), and anti-hepatitis (Ji et al., 2016). In addition, our previous studies have demonstrated its neuroprotective protective effect *in vitro* (Jiang et al., 2014; Liu et al., 2014). Further *in vivo* studies showed that the potential medicinal applications of CSA include, but are not limited to anti-depression and improving cognitive function (Wang L. S et al., 2019; Zhang et al., 2020). Some stilbenes such as resveratrol, having similar chemical structures compared with CSA, protects against APAP-induced liver and kidney injury (Du et al., 2015; Dallak et al., 2020). However, whether CSA alleviates APAP-induced hepatotoxicity and how CSA exerts its hepatoprotective activity is unknown. Data presented in this report suggest that CSA protects against APAP-induced liver injury through enhancing mitochondrial quality control, which is mediated by increased Sestrin2/AMPK signaling pathway.

## Materials and Methods

### Mice and Drug Treatments

Eight-week-old male C57BL/6 N mice (20–24 g) were purchased from Beijing Vital River Laboratory Animal Technology Co., Ltd. The mice were housed in plastic cages with corncob bedding, and maintained at a constant temperature (23 ± 2°C), humidity (55 ± 5%) and 12-h light/dark cycle (lights on at 8:00–20:00). They had free access to water and standard feed. The Animal Ethics Committee at the Institute of Medicinal Plant Development approved all procedures performed on animals (license number. SLXD-20191129002).

**FIGURE 1:**
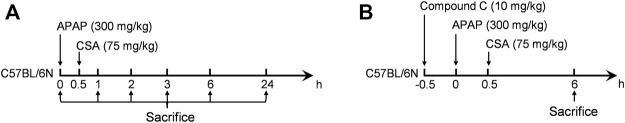
Experimental design. Mice were fasted overnight for 16 h before intraperitoneally (i.p.) injected with APAP. Blood and liver tissues were collected at the time point of sacrifice. **(A)** The time-course experiment. **(B)** The experiment for the investigation of AMPK.

After 1 week of acclimatization, the mice were randomly divided into eleven groups by body weight: vehicle control group, in which mice were injected with normal saline; five APAP-treated alone groups (300 mg/kg, i. p.); and five APAP + CSA groups, in which CSA (75 mg/kg) was orally administered to mice 0.5 h following APAP overdose. The blood samples and liver tissues were collected at 0 (vehicle control group), 1, 2, 3, 6, and 24 h after APAP injection respectively (Figure 1A). To study the role of AMPK in the protective effect of CSA, four groups of C57BL/6 N mice were used (*n* = 5–6/group): the normal saline group, APAP alone group, APAP + Compound C group and APAP + Compound C + CSA group. AMPK inhibitor Compound C dihydrochloride (MCE, HY-13418) was injected intraperitoneally to mice in a single dose of 10 mg/kg 0.5 h prior to APAP. CSA (75 mg/kg) was orally administered to mice 0.5 h following APAP overdose. The blood and liver tissues were collected 6 h after APAP overdose (Figure 1B).

### Measurement of Alanine Aminotransferase and Aspartate Aminotransferase

At 0, 6 and 24 h after APAP injection, the mice in different groups were weighed and anesthetized with pentobarbital sodium (65 mg/kg, i. p.). Subsequently, the blood samples were collected from the abdominal aorta and stored at 4°C for 2 h, then centrifuged at 1,000×*g* for 15 min to yield serum. The levels of alanine aminotransferase (ALT) and aspartate aminotransferase (AST) in the serum were measured on Beckman Coulter AU480 fully automated biochemical analyzer (Brea, CA, United States) with matched reagent kits (Biosino Bio, 1,00,020,003 and 1,00,020,013, Beijing, China).

### Histological Analysis

After collecting the blood samples, mice were euthanized by cervical dislocation. The livers were excised quickly and washed with cold phosphate-buffered saline. Sections of approximately 3–5 mm were fixed in 4% paraformaldehyde solution at room temperature for 24 h and embedded in paraffin. Tissues were manually processed with a microtome to obtain 5 μm thickness paraffin sections, followed with hematoxylin and eosin (H&E) staining for histological analysis. Terminal deoxynucleotidyl transferase-mediated dUTP Nick End Labeling (TUNEL) assay was performed to examine the apoptosis in mouse livers, according to the manufacturer’s instructions (Solarbio, T2190, Beijing, China). For immunohistochemical analyses, paraffin-embedded sections were stained with an anti-3-Nitrotyrosine antibody (Abcam Cat# ab53232, RRID:AB_869927) at a 1:250 dilution followed by heat-induced epitope retrieval and visualized by 3, 3′-Diaminobenzidine (DAB). For immunofluorescence analysis, the sections were stained with an anti-LC3 (ABclonal Cat# A19665, RRID:AB_2862723) antibody at a dilution of 1:200, and nuclei were counterstained with 4′, 6-diamidino-2-phenylindole dihydrochloride (Solarbio, C0065, Beijing, China) to show all nuclei before being mounted. The number of cells was counted manually.

### Primary Hepatocytes Culture and Treatments

Cell culture plates were coated with 0.5 mg/ml rat tail tendon collagen type Ⅰ (Solarbio, C8062, Beijing, China) overnight at 4°C and washed with PBS for three times before cell seeding. Murine hepatocytes were isolated from C57BL/6 N mice by a retrograde perfusion of liver with 30–40 ml HBSS (Gibco, 14175–095) containing 0.5 mM EGTA (Sigma, 03777), followed with about 40 ml low glucose DMEM containing 100U/mL collagenase type IV (Gibco, 17104–019). Hepatocytes were cultured in DMEM with 10% fetal bovine serum for 4 h for attachment. Then the medium was replaced with hepatocyte culture medium (LONZA, CC-3199 and CC-4182). The hepatocytes were treated with APAP (5 and 10 mM) in the absence or presence of CSA (50 μM) or chloroquine (20 μM) for 24 h. Cells were double stained with calcein and propidium iodide (Beyotime Biotech, C2015). After incubation at 37°C for 30 min, the fluorescence intensity was detected by Tecan’s Spark multimode reader to assess the percentage of cell death. For representative images, hepatocytes were stained with propidium iodide and Hoechst 33258 (10 μg/ml) for 30 min followed by fluorescence microscopy.

### Quantitative Real-Time PCR

Total RNA was extracted from liver tissues using TRIzon regent (CWbiotech, CW0580, Beijing, China). The cDNA libraries were synthesized using TransScript^®^ SuperMix kits for qPCR (TransGene, AT341). qPCR was performed using the TransStart^®^ Top Green SuperMix (TransGene, AQ131) on a Roche LightCycler 96 instrument. Ct values were normalized to an internal control of *GAPDH* (Sangon Biotech, B661304, Shanghai, China). The primers used in qPCR are shown in Table 1.

**TABLE 1:** Sequences of real time PCR primers.

Gene	Forward	Reverse
*IFN-γ*	CTT​GAA​AGA​CAA​TCA​GGC​CAT​C	CTT​GGC​AAT​ACT​CAT​GAA​TGC​A
*TNF*	ATG​TCT​CAG​CCT​CTT​CTC​ATT​C	GCT​TGT​CAC​TCG​AAT​TTT​GAG​A
*IL-1β*	CAC​TAC​AGG​CTC​CGA​GAT​GAA​CAA​C	TGT​CGT​TGC​TTG​GTT​CTC​CTT​GTA​C
*iNOS*	ATC​TTG​GAG​CGA​GTT​GTG​GAT​TGT​C	TCG​TAA​TGT​CCA​GGA​AGT​AGG​TGA​GG
*GAPDH*	GGT​TGT​CTC​CTG​CGA​CTT​CA	TGG​TCC​AGG​GTT​TCT​TAC​TCC

### Immunoblot Analysis

About 20 mg of frozen liver tissues were lysed and homogenized in 400 μl RIPA buffer with 1% protease and phosphatase inhibitor cocktail (100×). After centrifuging at 14000×*g* for 20 min at 4°C, protein concentration of the supernatant was determined by the BCA protein assay kit (Solarbio, PC0020, Beijing, China) and was normalized to a final concentration of 5 μg/μl. Ten microliters denatured proteins were separated by 10% or 12% sodium dodecyl sulfate-polyacrylamide gel electrophoresis (SDS-PAGE) and electrophoretically transferred to nitrocellulose membranes (Pall Corporation, 0.2 μm). The membranes were blocked with 5% nonfat milk in TBST buffer for 1 h at room temperature and incubated with primary antibodies specific for STING (ABclonal Cat# A3575, RRID:AB_2765161), p-IκBα (ABclonal Cat# AP0707, RRID:AB_2863811), IκBα (ABclonal Cat# A11397, RRID:AB_2861556), NLRP3 (Cell Signaling Technology Cat# 15101, RRID:AB_2722591), p-p65 (Cell Signaling Technology Cat# 3033, RRID:AB_331284), p65 (Proteintech Cat# 10745-1-AP, RRID:AB_2178878), CYP2E1 (ABclonal Cat# A2160, RRID:AB_2764178), p-JNK (Cell Signaling Technology Cat# 9251, RRID:AB_331659), JNK (ABclonal Cat# A4867, RRID:AB_2863367), 3-Nitrotyrosine (Abcam Cat# ab53232, RRID:AB_869927), PGC-1α (Abcam Cat# ab54481, RRID:AB_881987), TFAM (Abcam, Cat# ab252432), PINK1 (ABclonal Cat# A7131, RRID:AB_2767686), LC3 (ABclonal Cat# A19665, RRID:AB_2862723), p62 (Proteintech Cat# 18420-1-AP, RRID:AB_10694431), Parkin (ABclonal Cat# A0968, RRID:AB_2757487), p-AMPKα (Cell Signaling Technology Cat# 2535, RRID:AB_331250), AMPKα (Abcam, ab207442), CAMKK2 (Proteintech Cat# 11549-1-AP, RRID:AB_2259441), Sestrin2 (Proteintech Cat# 10795-1-AP, RRID:AB_2185480), LKB1 (ABclonal Cat# A2122, RRID:AB_2764141), LC3B (Cell Signaling Technology Cat# 3868, RRID:AB_2137707), VDAC1 (Proteintech Cat# 55259-1-AP, RRID:AB_10837225) and GAPDH (ABclonal Cat# AC001, RRID:AB_2619673) at 4°C shaking overnight. Wash the membrane three times for 5 min each with TBST, followed with incubating in the diluted secondary antibody (1:5,000) on a shaker for 1 h at room temperature. Protein bands were detected by an enhanced chemiluminescent reagent kit (Beyotime Biotech, P0018FS, Shanghai, China) using a ChemiDocTM Imager (Bio-Rad, Hercules, CA, United States).

### GSH Measurement

Mouse liver samples that were collected at 0, 1, 2, 3, 6 h after APAP injection were analyzed for reduced glutathione levels using the colorimetric reagent kit (Nanjing Jiancheng, A006-2-1, Nanjing, China).

### ATP Measurement

Liver tissue samples that were collected at 6 h after APAP injection were homogenized in lysis buffer, and centrifuged at 12000×*g*, 4°C for 5 min. The supernatant and various concentrations of ATP standard solutions were incubated with luciferase reagent according to the manufacturer’s instruction (Beyotime Biotech, S0026, Shanghai, China). Counts per minute (CPM) of each sample were analyzed using the MicroBeta2 Microplate Counters (PerkinElmer, Waltham, MA, United States) for calculating ATP levels.

### Measurement of Mitochondrial Respiratory Complex I Activity

Mitochondrial respiratory complex I activities of mouse liver samples were assayed using the commercial reagent kit according to the manufacture’s protocol (Solarbio, BC0515, Beijing, China). After adding submitochondrial particles, the decrease in absorbance due to oxidation of NADH to NAD^+^ at 340 nm for 2 min were measured, and then normalized to protein concentrations to calculate the complex I activities.

### Subcellular Fractionation for Mitochondria From Liver Tissue

Mitochondria were isolated from murine livers using a tissue mitochondria extraction kit from Solarbio (SM0020, Beijing, China) as previously described (Yan et al., 2018b). The isolated mitochondria were lysed in RIPA buffer and subjected to Western blot analysis.

### Transmission Electron Microscopy

Liver tissues were processed as described previously (Yan et al., 2021). The ultrathin sections (70 nm thick) were double stained with uranyl acetate and lead citrate, and viewed under a transmission electron microscope (HT7700, Tokyo, Japan) at 80.0 kV. Normal mitochondria show double-membraned, oval-shaped structure, with cristae derived from inner membrane. Mitophagosome is characterized by a double membrane structure that is engulfing damaged mitochondria. Mitolysosome refers to the product of a mitophagosome fused with a lysosome. Both mitophagosome and mitolysosome are defined as mitophagic vacuoles (Eid et al., 2019; Klionsky et al., 2021).

### Statistical Analysis

The data were presented as mean ± standard errors of mean (SEM). GraphPad Prism software (version 9.1.0) was used to analyze the data derived from the experiments and draw graphs. Firstly, the normality of the data was tested by Shapiro-Wilk test. Statistical comparisons were performed using unpaired two-tailed Student’s *t* test, one-way or two-way analysis of variance (ANOVA) followed by post hoc Dunnett’s test where appropriate.

## Results

### CSA Alleviates APAP-Induced Hepatotoxicity *in Vivo* and *in Vitro*

**FIGURE 2:**
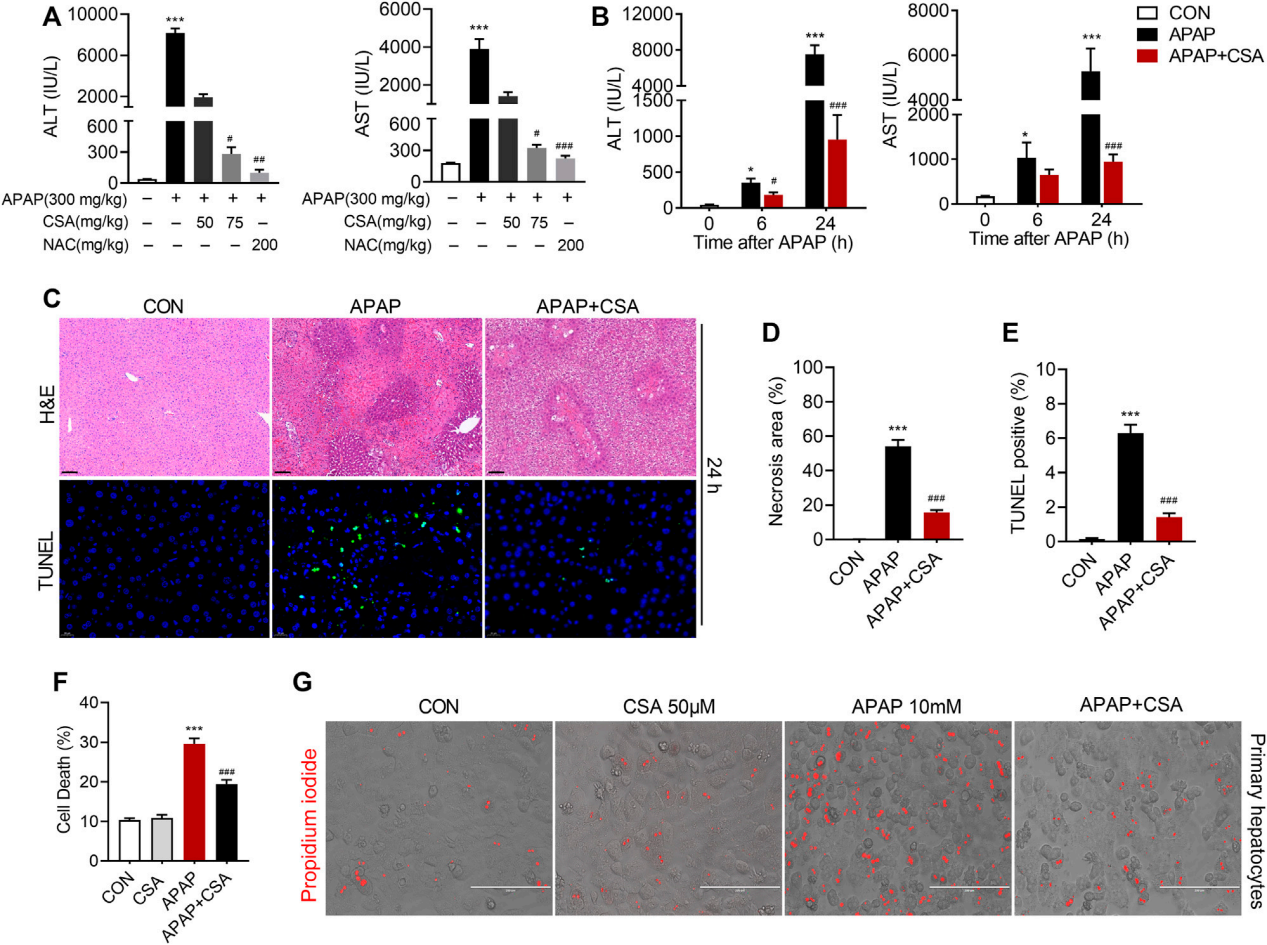
Cajaninstilbene acid (CSA) protects against APAP-induced liver injury. CSA (50 and 75 mg/kg, p. o.) or NAC (200 mg/kg) was administered to mice 0.5 h after APAP (300 mg/kg, i. p.) (*n* = 6 mice per group). **(A)** Serum ALT and AST levels were measured 24 h after APAP. CSA (75 mg/kg, p. o.) was administered to mice 0.5 h after APAP (300 mg/kg, i. p.) (*n* = 8–10 mice per group). **(B)** Serum ALT and AST levels were measured at 6 and 24 h after APAP. **(C)** Representative images of H&E staining (upper panel, scale bar = 100 μm) and TUNEL staining (lower panel, scale bar = 20 μm) at 24 h after APAP overdose. **(D)** Quantification of the centrilobular necrotic areas. **(E)** Quantification of the TUNEL-positive hepatocyte nucleis. Primary hepatocytes were treated with APAP (10 mM) for 24 h in the presence or absence of CSA (50 μM) **(F)** Cell death were assessed by co-staining with calcein and propidium iodide. **(G)** Representative phase-contrast images overlaid with propidium iodide signals. ^*^ *p* < 0.05, ^***^ *p* < 0.001 compared with the vehicle control group; ^#^ *p* < 0.05, ^###^ *p* < 0.001 compared with the corresponding APAP-treated alone group.

To identify the therapeutic effects of CSA on APAP-induced acute liver injury, different doses of CSA (50 and 75 mg/kg) was administered to C57BL/6 N mice 0.5 h after APAP treatment. Though not as effective as the standard antidote NAC, CSA protected mice from APAP-induced liver injury in a dose-dependent manner, which was evidenced by progressively decreased serum ALT and AST levels (Figure 2A). In the time course experiments, obvious liver injury was detected in mice treated with 300 mg/kg APAP alone 6 h after intoxication, as evidenced by significantly elevated serum ALT and AST levels (Figure 2B). As the liver damage progressed, the levels of ALT and AST peaked at 24 h (Figure 2B). To further determine the efficacy of CSA in treating APAP-induced liver injury, a single dose of this compound (75 mg/kg) was orally given to mice 0.5 h following APAP overdose. Mice treated with CSA showed reduced ALT and AST levels by 47 and 37% at 6 h, and by 87 and 82% at 24 h respectively (Figure 2B). In line with this, the histological analysis revealed that the extent of APAP-induced necrosis and apoptosis in zone 3 of the liver lobules was dramatically attenuated by the CSA (Figure 2C). Statistical analysis further showed that the necrosis area and TUNEL positive cells in mice treated with CSA significantly decreased (Figures 2D,E), when compared with the group of APAP-treated alone. To confirm the direct effect of CSA on hepatocytes, primary mouse hepatocytes cultured *in vitro* were cotreated with CSA and APAP. We found that CSA substantially reduced APAP-induced cell death after APAP treatment for 24 h (Figures 2F,G). Thus, these results demonstrate that CSA can effectively protect against APAP-induced liver injury both *in vivo* and *in vitro* models.

### CSA Attenuates Hepatic Inflammation Induced by APAP Intoxication

**FIGURE 3:**
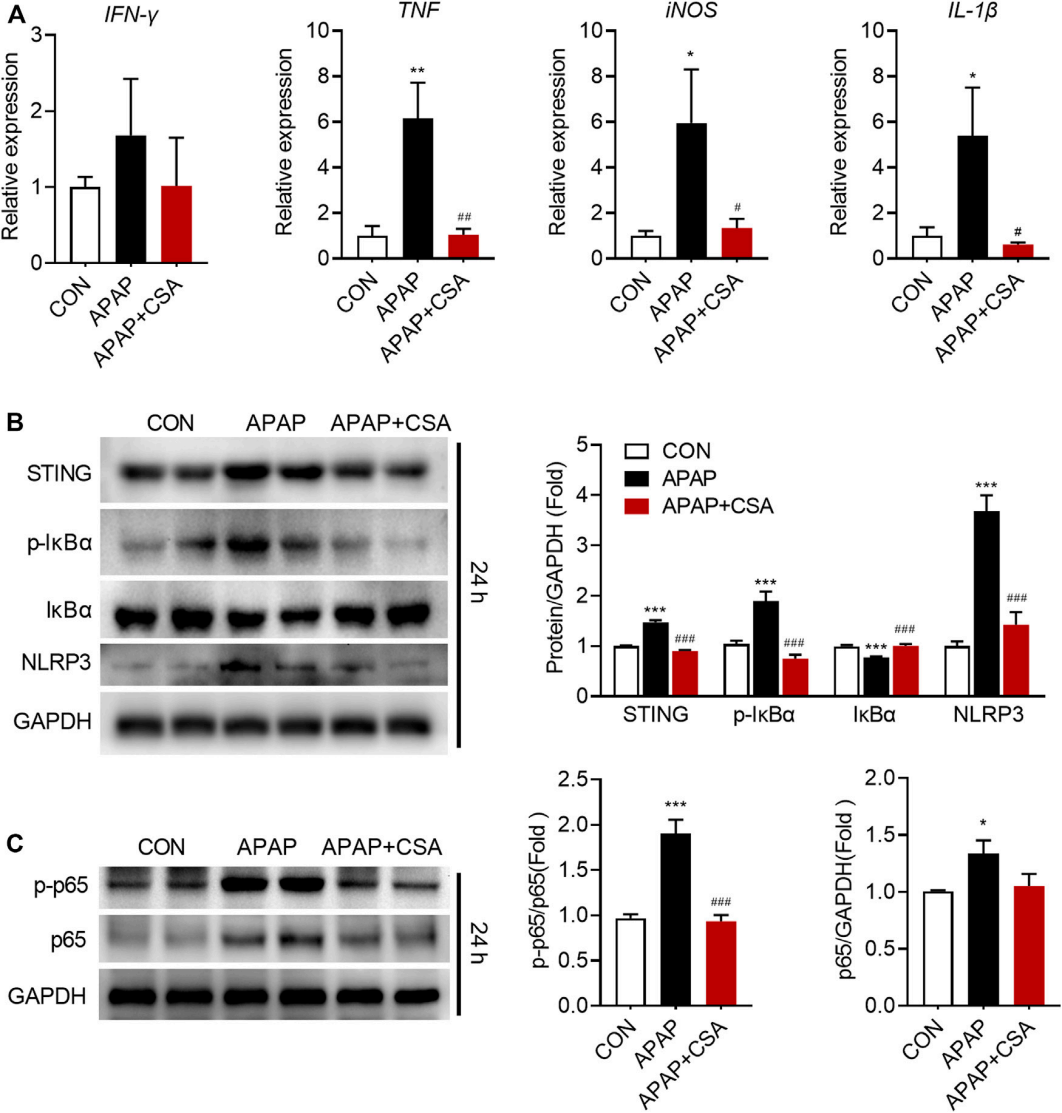
Cajaninstilbene acid (CSA) reduces the inflammatory response in the liver following APAP overdose. Animal experiment was identical to that in Figure 1, and livers collected at 24 h after APAP administration were analyzed. **(A)** Gene expressions in the liver at 24 h after APAP overdose were measured by qPCR, presented relative to *GAPDH* (*n* = 6–8/group). **(B,C)** Total protein of liver tissue was analyzed by immunoblotting. The graph shows results of densitometric analyses of STING, IκB, p-IκB, NLRP3, p65 relative to GAPDH respectively, and p-p65 relative to total p65 (*n* = 6/group). ^*^ *p* < 0.05, ^**^ *p* < 0.01, ^***^ *p* < 0.001 compared with the vehicle control group; ^#^ *p* < 0.05, ^##^ *p* < 0.01, ^###^ *p* < 0.001 compared with the corresponding APAP-treated group.

APAP-induced liver injury triggers the innate immune system that has been implicated in the progression of tissue damage. We hence examined that whether CSA modulated the cascade of the immune responses. As expected, hepatic mRNA expression of *IFN-γ*, *TNF*, *iNOS* and *IL-1β* markedly increased following APAP overdose at 24 h (Figure 3A), suggesting a pro-inflammatory response. In contrast, CSA treatment reduced several of these genes induced by APAP, including *TNF*, *iNOS* and *IL-1β* (Figure 3A). To further confirm the inhibitory action of CSA on the inflammatory response, several associated signaling pathways were examined by western blot. In agreement with the reduced *IFN-γ* mRNA levels, its upstream regulatory protein STING significantly decreased upon CSA treatment (Figure 3B). Similarly, APAP overdose induced phosphorylation of IκBα (Figure 3B), leading to the proteasome-mediated degradation that results in the release and nuclear translocation of NF-κB. The expression of p65, a subunit of NF-κB, and the phosphorylation of p65 at Ser536, which promotes its nuclear translocation, were all upregulated after APAP overdose (Figure 3C). As indicated by immunoblotting analysis, the key activator of inflammation NF-κB induced NLRP3 expression (Figure 3B), which recruits caspase-1 to form inflammasome and cleaves pro-IL-1β into mature IL-Iβ. CSA treatment abolished the activation of STING, NLRP3 and the phosphorylation of IκBα and p65 induced by APAP overdose (Figures 3B,C).

### CSA has No Effect on the Metabolic Activation of APAP

**FIGURE 4:**
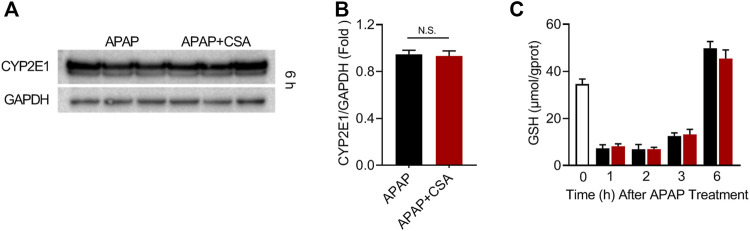
Cajaninstilbene acid (CSA) does not inhibit the metabolic activation of APAP. Animal experiment was identical to that in Figure 1, and liver tissues were excised at 1, 2, 3, 6 h after APAP administration respectively. **(A)** Western blot analysis of whole liver homogenate for CYP2E1 and **(B)** the corresponding densitometry normalized to GAPDH (*n* = 6/group). **(C)** Hepatic GSH levels at 0, 1, 2, 3 and 6 h after APAP (*n* = 4–6/group). N.S, nonsignificant.

To decipher the therapeutic mechanisms of CSA, cytochrome P450 (CYP) 2E1, a major pro-toxic metabolic enzyme for APAP, was determined in the liver of mice 6 h after APAP overdose. The data showed that CSA did not affect the expression of CYP2E1 in comparison to the APAP group (Figures 4A,B). The kinetics of hepatic GSH reflected APAP-protein adducts formation. Hence, we observed a significant decline of GSH in the whole-liver tissue at 1, 2 and 3 h after APAP overdose. Gradually, hepatic GSH levels spontaneously recovered, even with an additional increase in contrast to the baseline (Figure 4C). Mice treated with CSA exhibited equivalent levels of GSH compared with the corresponding APAP group (Figure 4C), suggesting that GSH depletion or recovery was not affected by CSA. Collectively, these results demonstrated that CSA did not interfere with APAP metabolism. In other words, protection by CSA against APAP hepatotoxicity did not result from inhibiting APAP metabolic activation.

### CSA Attenuates APAP-Induced Oxidative Stress and Mitochondrial Dysfunction

**FIGURE 5:**
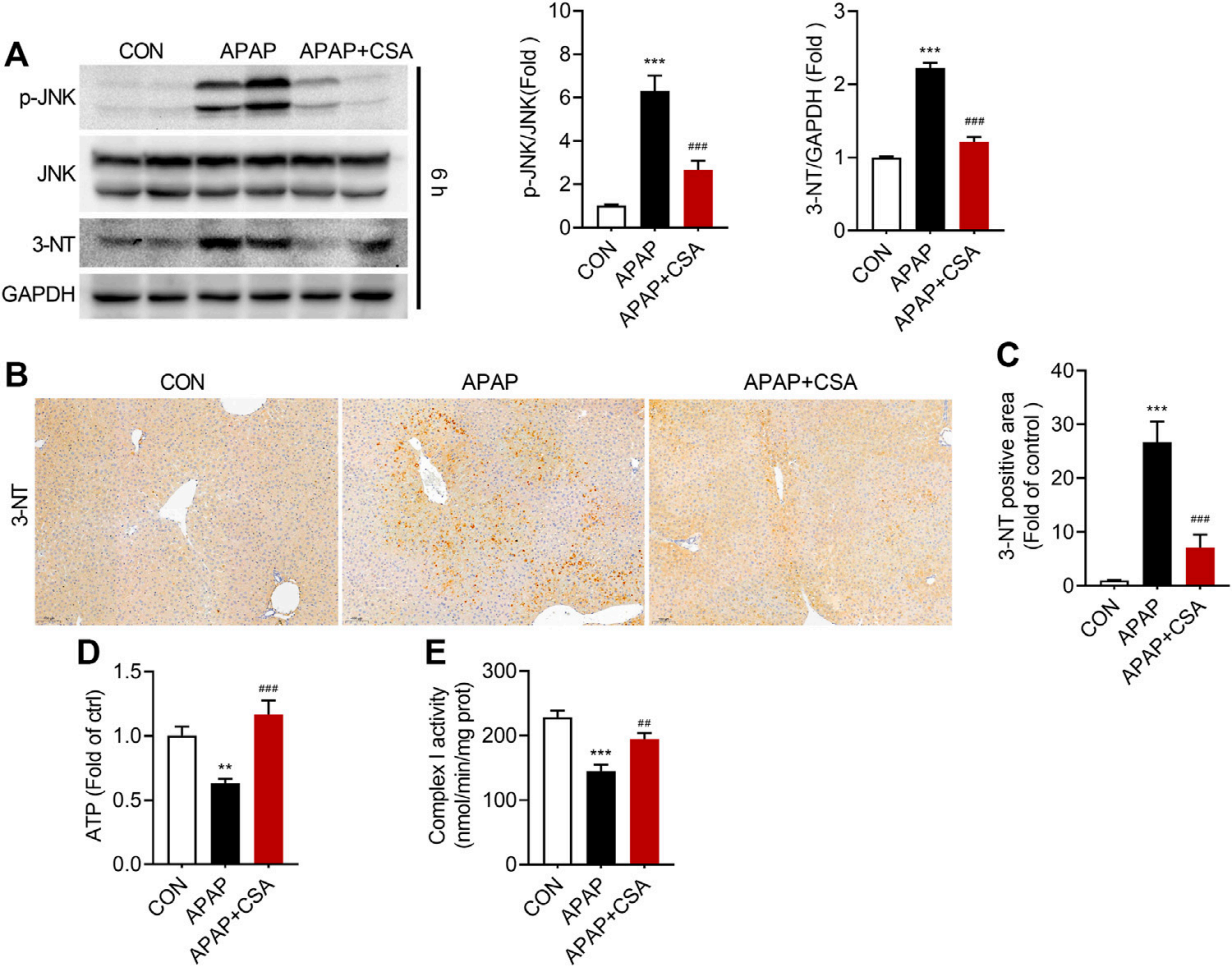
Cajaninstilbene acid (CSA) inhibits JNK signaling pathway in APAP-induced liver injury. Animal experiment was identical to that in Figure 1, and liver tissues were excised at 6 h after APAP overdose. **(A)** Western blot analysis of whole liver homogenate for p-JNK, JNK, 3-Nitrotyrosine (left panel) and the corresponding densitometry normalized to GAPDH (right panel) (*n* = 6–7/group). **(B)** Representative immunostaining of 3-Nitrotyrosine in liver tissues (scale bar = 100 μm). **(C)** Quantification with ImageJ of 3-Nitrotyrosine positive areas (*n* = 6/group). **(D)** Mouse liver ATP levels. **(E)** Enzyme activity of mitochondrial respiratory complex I. ^**^ *p* < 0.01, ^***^ *p* < 0.001 compared with the vehicle control group; ^##^ *p* < 0.01, ^###^ *p* < 0.001 compared with the corresponding APAP-treated alone group.

Mitochondrial oxidative stress plays the central role in AILI. In accordance with this, we detected an increase in phosphorylation of Jun NH2-terminal kinase (JNK), an important signaling molecule involved in the pathological process of AILI, and 3-Nitrotyrosine, the oxidation product formed exclusively in mitochondria in response to p-JNK (Figure 5A). CSA treatment blocked the significant elevation of p-JNK and 3-Nitrotyrosine, as evidenced by western blot analysis. To verify that CSA suppresses APAP-induced oxidative stress, we analyzed 3-Nitrotyrosine by immunohistochemistry. This biomarker of oxidative damage was predominantly observed and accumulated in the centrilobular region of the liver (zone 3) (Figure 5B), the area where APAP-induced liver damage is prone to occur. The staining intensity and 3-Nitrotyrosine positive area robustly decreased after CSA treatment (Figures 5B,C), suggesting the alleviation of oxidative stress caused directly by APAP overdose. As a consequence, APAP-induced decreases in ATP and mitochondrial respiratory complex I activity were restored by CSA (Figures 5D,E). Combined, the data demonstrate that CSA alleviates APAP-induced mitochondrial damage.

### CSA Enhances Mitochondrial Quality Control After APAP Overdose

**FIGURE 6:**
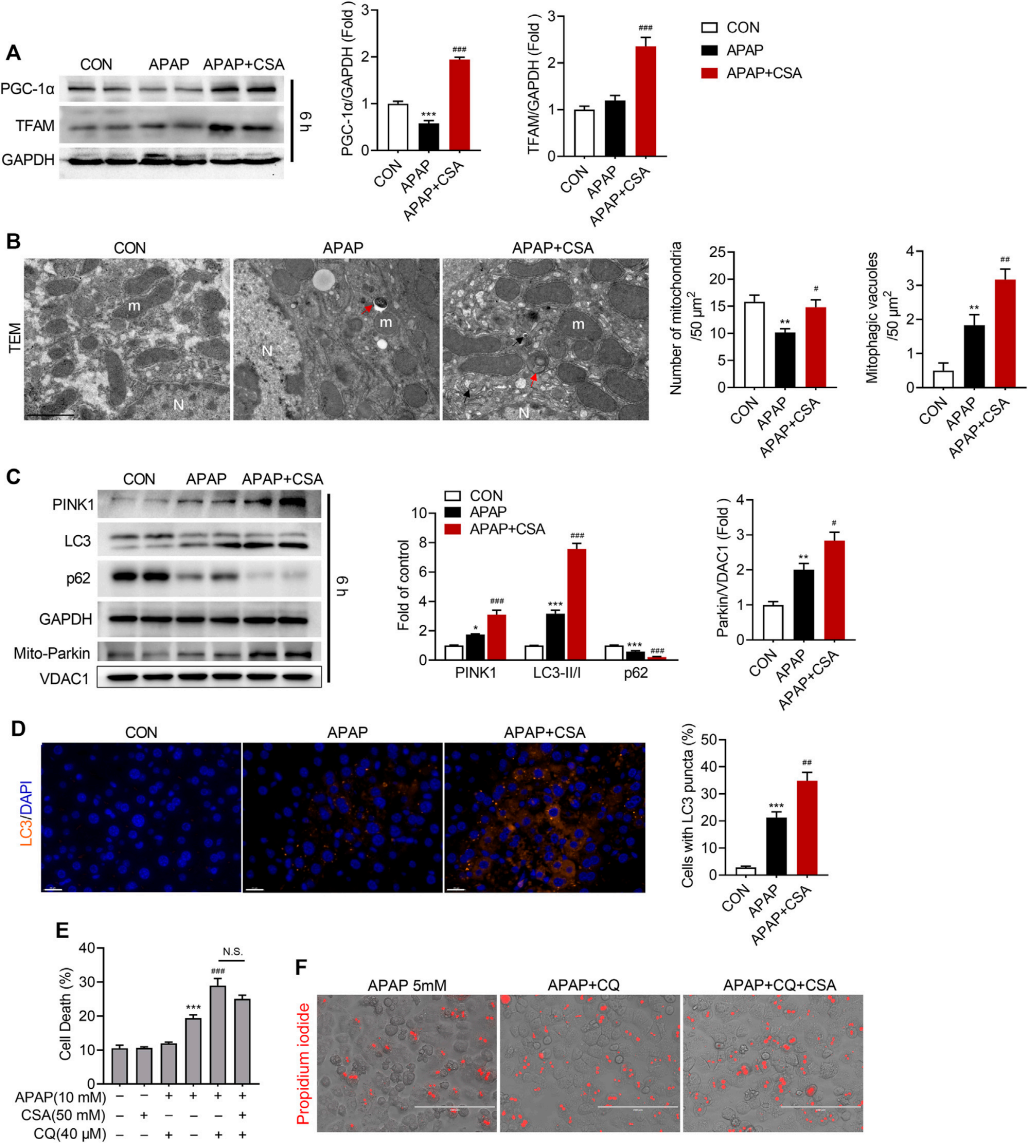
Cajaninstilbene acid (CSA) promotes mitochondrial biogenesis and mitophagy in APAP-induced liver injury. Animal experiment was identical to that in Figure 1, and liver tissues were excised at 6 h after APAP overdose. **(A)** Western blot analysis of whole liver homogenate for PGC-1α, TFAM (left panel) and the corresponding densitometry normalized to GAPDH (right panel) (*n* = 6/group). **(B)** Representative transmission electron microscopy (TEM) images of hepatocytes in the liver (scale bar = 1 μm). Red arrows denote for the mitophagosomes. Black arrows mark mitolysosomes. m = mitochondria, N = nucleus. The number of mitochondria and mitophagic vacuoles (mitophagosomes and mitolysosomes) was quantified from six cells. **(C)** Western blot analysis of whole liver homogenate for PINK1, LC3, p62 and mitochondrial extracts for Parkin and VDAC1 (left panel). The graph shows results of densitometric analyses (*n* = 5–6/group). **(D)** Representative images of the dual immunofluorescence staining for LC3 (orange) and 4′, 6-diamidino-2-phenylindole (DAPI, blue) (left panel, scale bar = 20 μm), and quantification of hepatocytes with LC3 puncta (right panel, *n* = 6/group). Primary hepatocytes were treated with APAP (5 mM) for 24 h in the presence or absence of CQ (20 μM) or CSA (50 μM). **(E)** Cell death were assessed by co-staining with calcein and propidium iodide. **(F)** Representative phase-contrast images overlaid with propidium iodide signals. ^*^ *p* < 0.05, ^**^ *p* < 0.001, ^***^ *p* < 0.001 compared with the vehicle control; ^#^ *p* < 0.05, ^##^ *p* < 0.01, ^###^ *p* < 0.001 compared with the corresponding APAP-treated group.

To determine how CSA attenuates APAP-induced oxidative stress, we examined processes that are vital for mitochondrial function and quality control. As previously reported (Du et al., 2017), PGC-1α decreased when detected at 6 h after APAP overdose, suggesting that mitochondrial biogenesis is disturbed by APAP (Figure 6A) at this time point. However, CSA promoted the expression of PGC-1α when compared with APAP-treated alone and even the vehicle control (Figure 6A). The expression of the mitochondrial transcription factor TFAM, downstream of PGC-1α, was also upregulated by CSA (Figure 6A). Additionally, TEM showed an increased number of mitochondria with normal morphology in mice-treated with CSA (Figure 6B). Unlike mitochondrial biogenesis, mitophagy was activated in the mice liver 6 h after APAP overdose, as indicated by increased levels of PINK1, LC3-II/LC3-Ⅰ and mitochondrial Parkin (Figure 6C). CSA treatment enhanced the expression of mitophagy protein PINK1, as well as LC3-II (Figure 6C). Mitochondrial translocation of Parkin was further induced by CSA, implying more robust mitophagy. In line with the changes detected by western blot, the number of mitophagic vacuoles (mitophagosomes and mitolysosomes) and LC3 puncta per hepatocyte increased after CSA treatment, when compared with that of APAP-treated alone, as illustrated by TEM (Figure 6B) and immunohistochemical analysis (Figure 6D). Moreover, the continuous decrease in p62 expression verified the unobstructed autophagic flux (Figure 6C), ruling out the possibility that the increase in PINK1, Parkin and LC3-II was caused by blocked autophagy (Yan et al., 2021). To test the involvement of mitophagy, primary hepatocytes were treated with CQ to block the autophagy before CSA treatment. The reduction in cell death caused by CSA was partially reversed by CQ (Figures 6E,F). Together, these data suggest that CSA enhances mitochondrial quality control by promoting biogenesis and mitophagy, which contributes to the therapeutic effect of CSA.

### CSA Promotes Sestrin2/AMPK Activation in APAP-Induced Liver Injury

**FIGURE 7:**
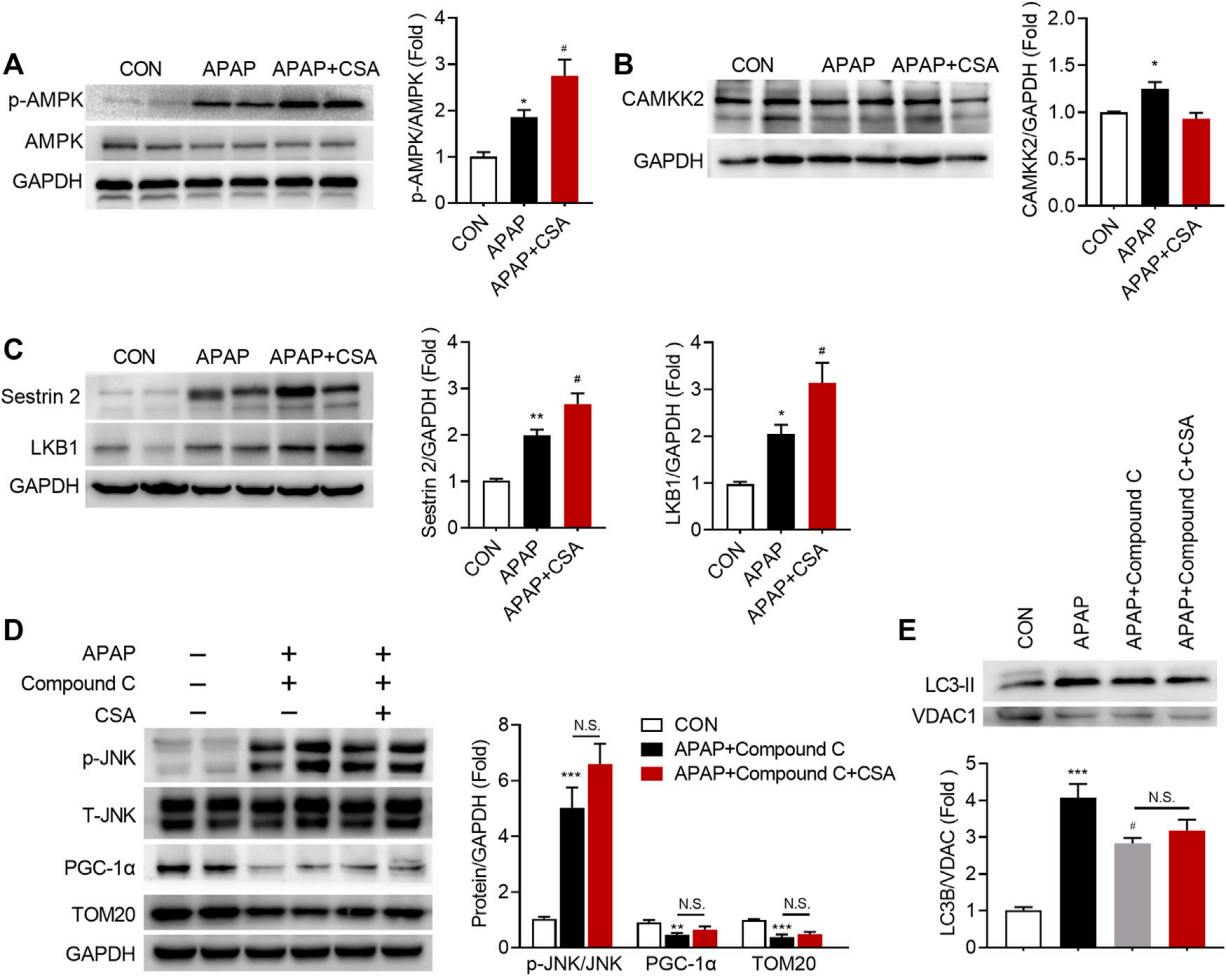
APAP-induced Sestrin2/AMPK activation is enhanced in the liver of mice treated with cajaninstilbene acid (CSA). **(A–C)** Animal experiment was identical to that in Figure 1, and liver tissues were collected at 6 h after APAP overdose. Immunoblot analysis of phosphorylated (p-) or total protein in lysates of total liver extracts. The graphs show results of densitometric analyses of p-AMPK relative to total AMPK, CAMKK2, Sestrin2, LKB1 relative to GAPDH respectively (*n* = 6/group). **(D,E)** Compound C (10 mg/kg) was injected to mice 0.5 h prior to APAP, followed by CSA (75 mg/kg) administration. Liver tissues were collected at 6 h after APAP overdose. **(D)** Immunoblot analysis of whole liver homogenate. The graphs show results of densitometric analyses of p-JNK relative to total JNK, PGC-1α, TOM20 relative to GAPDH respectively. **(E)** Immunoblot analysis of mitochondrial LC3 II and the corresponding densitometry normalized to VDAC1. ^*^ *p* < 0.05, ^**^ *p* < 0.001, ^***^ *p* < 0.001 compared with the vehicle control; ^#^ *p* < 0.05, ^##^ *p* < 0.01, ^###^ *p* < 0.001 compared with the corresponding APAP-treated group. N.S, nonsignificant.

To discover the molecular mechanisms that drive CSA to benefit mitochondrial function, we first tested the phosphorylation status of AMPK at Thr172, as AMPK acts as the guardian of mitochondrial homeostasis (Herzig and Shaw, 2018). APAP overdose caused an increase in phosphorylation of AMPK, which was further enhanced by CSA treatment (Figure 7A). Next, we aimed to explore the upstream signals that might account for the activation of AMPK. The expression of CAMKK2, an important regulator of AMPK activity, though significantly increased following APAP overdose, decreased after CSA treatment and was comparable with the vehicle control group (Figure 7B). By contrast, the kinase LKB1, which is the major regulator of AMPK activation, was upregulated following APAP challenge (Figure 7C). And similar to p-AMPK, its expression was further elevated after CSA intervention (Figure 7C). In parallel, Sestrin2, a scaffold protein that interacts with LKB1 to activate AMPK, also increased in CSA-treated group when compared with APAP administration alone (Figure 7C). Inhibiting AMPK activation with compound C prevented the increase of PGC-1α and LC3-II in the mitochondria after CSA administration (Figures 7D,E), suggesting that AMPK mediates the CSA-induced mitochondrial quality control enhancement. This was further confirmed by the mitochondrial marker protein TOM20 (Figure 7D), which showed no significant changes in mice with and without CSA after inhibition of AMPK. Furthermore, suppression of p-JNK caused by CSA was also blocked by compound C pretreatment (Figure 7D). Collectively, our data demonstrate that although CAMKK2 is dispensable, Sestrin2/LKB1/AMPK appears to be responsible for CSA-induced enhancement in mitochondrial quality control.

## Discussion

As early as the 1960s, clinical cases of APAP-associated liver injury began to appear (Thomson and Prescott, 1966). In the United States, AILI was fast becoming the leading cause of ALF and accounts for 46% of all the cases (Lee, 2017). In the United Kingdom and Europe, the percentage is between 30 and 50% (Bernal et al., 2010; Bernal and Wendon, 2013). Thus, the hepatotoxicity induced by APAP overdose attracted extensive attention. Although NAC was recommended by FDA to treat AILI, the narrow therapeutic window limits its usage in the late-presenting patients. In the present study, we found CSA, a stilbene compound isolated from pigeon pea, was protective in a murine model of AILI through mechanisms involved in Sestrin2/AMPK activation and the related mitochondrial quality control modulation.

After APAP overdose, histological changes including hepatocytes and organelle swelling, nucleolysis, membrane rupture and cell contents release are observed. Based on these morphological characteristics, APAP-induced cell death is typically termed programmed necrosis (Jaeschke et al., 2019). CSA markedly ameliorated APAP-induced necrosis, as evidenced by H&E staining. Despite controversy, other types of cell death such as apoptosis, ferroptosis were recently reported to be involved in AILI (Wang et al., 2019; Yamada et al., 2020). Although we observed very limited amounts of TUNEL-positive nucleus in mice liver of APAP overdose, the amounts decreased after CSA treatment. Due to the protective effect of CSA against APAP-induced different types of cell death, serum levels of ALT and AST, which indicates the extent of liver damage, markedly decreased in CSA group when compared with APAP-treated alone. Cell death triggered by APAP overdose is accompanied by the release of cellular contents, which includes mitochondrial DNA (mtDNA), high-mobility group box 1 protein (HMGB1, nuclear-binding protein), adenosine triphosphate (ATP), uric acid and nuclear DNA fragments, which are all potent damage associated molecular patterns (DAMPs) (Woolbright and Jaeschke, 2017). They bind to the Toll-like receptors (TLRs) and initiate a signaling cascade that increases gene expressions of several pro-inflammatory cytokines (e.g., TNF-α, IL-1β), in macrophages (Williams et al., 2011). Binding of the TLRs also induces NLRP3 inflammasome activation, possibly via the NF-κB signaling pathway. Consistent with this, APAP overdose transcriptionally activated *IFN-γ*, *TNF-α*, *iNOS* and *IL-1β* in the present study. The mRNA levels of these pro-inflammatory cytokines significantly decreased after CSA intervention. Though the upstream proteins that regulate the transcription of these genes were also inhibited by CSA, we cannot conclude that the protective effect of CSA against AILI resulted from the anti-inflammation action of CSA. Because the inhibitory effect of CSA on inflammation was observed at 24 h after APAP overdose, and this may be a consequence of early-stage impairment alleviation.

The aforementioned result raised a question as what was the underlying mechanism behind the protective effect of CSA against AILI. Because CSA was administered 0.5 h after APAP, when APAP has not been completely converted into NAPQI (McGill et al., 2013), we started the research from the metabolic activation phase of AILI. The results demonstrated that neither the expression of CYP2E1 nor the kinetics of GSH was altered by CSA, ruling out the possibility that the reduced liver damage was caused by the inhibition of APAP metabolism. Mitochondrial oxidative stress has been widely recognized as the key element in the injury phase of AILI, and acts as downstream of APAP metabolic bioactivation to promote liver injury (Du et al., 2016). GSH depletion leads to the release of electrons from the mitochondrial electron transport chain (ETC) and thus forming reactive oxygen species (ROS). JNK is activated in response to the ROS and then translocate to the mitochondrial outer membrane, where p-JNK binds to Sab, leading to a sustained p-JNK-Sab-ROS activation loop (Win et al., 2018). Notably, in the current study, CSA inhibited the APAP-induced prolonged JNK activation, which subsequently disrupted the above toxic loop and reduced the oxidative stress products.

Because mitochondria are the main target of NAPQI, mitochondrial quality control, including mitochondrial biogenesis and mitophagy, is becoming an important strategy to reduce ROS and limit tissue damage (Moles et al., 2018). In line with the previous studies (Du et al., 2017), though expression of PGC-1α was diminished at 6 h after APAP overdose, the expression of TFAM increased, suggesting biogenesis of new mitochondria. CSA enhanced this process, as evidenced by further increased PGC-1α and TFAM. As thus, the number of normal mitochondria dramatically increased when compared with APAP-treated alone group. Additionally, mitophagy that removes the damaged mitochondria was also promoted by CSA treatment. These results strongly suggest that CSA enhances mitochondrial quality control, thereby maintaining mitochondrial and cellular homeostasis. AMPK is considered a signal integration platform to guard mitochondrial health (Herzig and Shaw, 2018). Regarding the mechanisms of enhanced mitochondrial quality control, we found that the phosphorylation of AMPK was further elevated by CSA, which was consistent with the tendency of increased TFAM and PINK1. NAPQI is reported to interfere with the function of membrane proteins that maintain calcium (Ca^2+^) homeostasis, thus increasing intracellular Ca^2+^ concentrations (Nicotera et al., 1989). In this context, the expression of CAMKK2 increased in response to APAP overdose (Figure 7B), which possibly activated AMPK, as CaMMK2 is one of the upstream kinases that can activate AMPK. However, after CSA treatment, the expression of CAMKK2 decreased, strongly suggesting that other upstream kinases were involved in regulating AMPK. In this study, it appeared to be LKB1, the critical upstream kinase required for the activation of AMPK, which was further upregulated by CSA, along with the LKB1-AMPK scaffolding protein Sestrin2.

**FIGURE 8:**
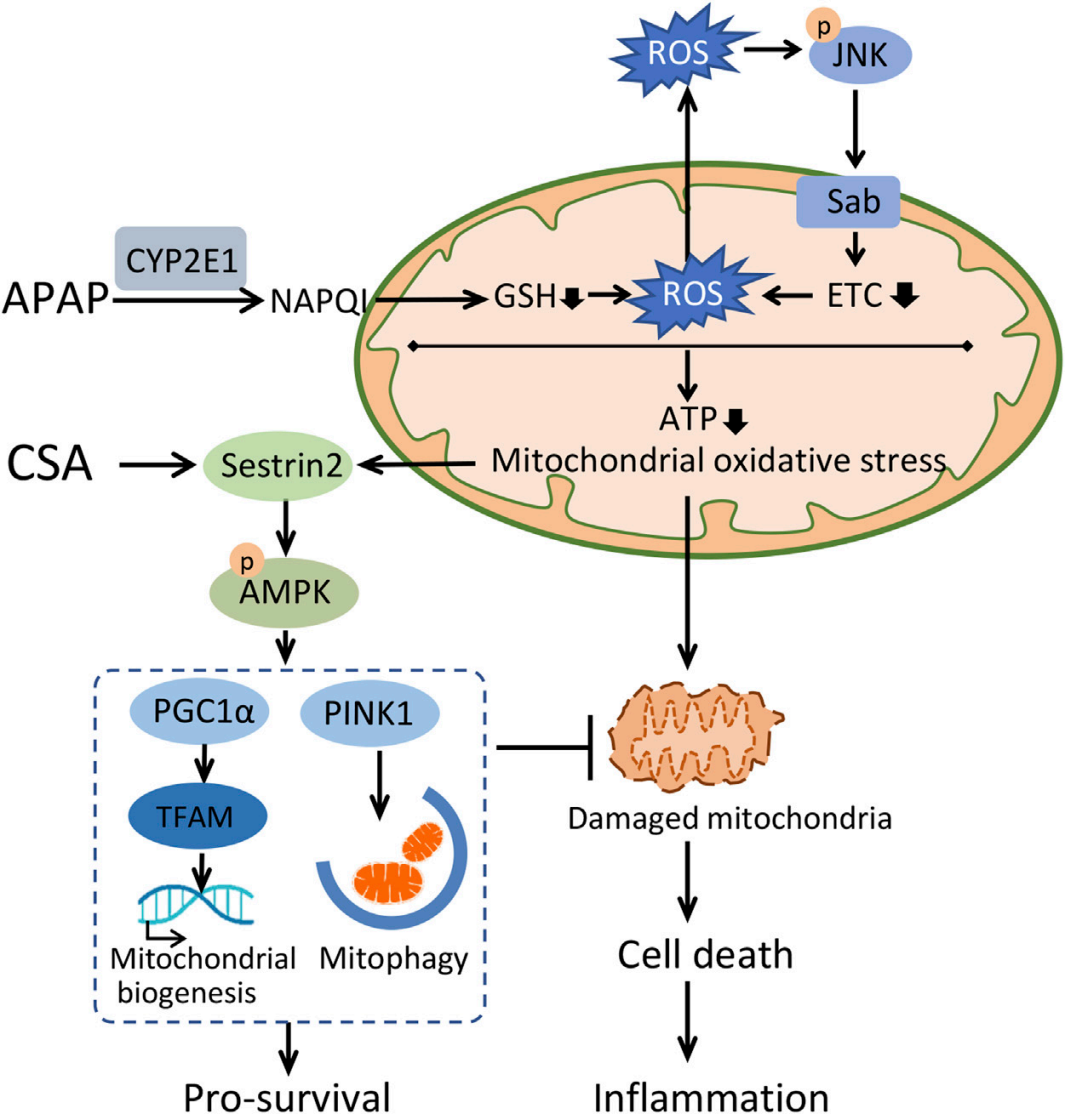
Schematic illustration of the mechanisms underlying therapeutic effect of cajaninstilbene acid (CSA) against APAP-induced liver injury. APAP overdose causes accumulation of N-acetyl-p-benzoquinone imine (NAPQI) predominantly via CYP2E1. Excess NAPQI depletes GSH, leading to the sustained activation of JNK-Sab-ROS loop and mitochondrial dysfunction, characterized by a loss of efficiency in the electron transport chain (ETC) and reductions in the synthesis of ATP. This ultimately results into hepatocyte cell death, which triggers the innate immune system. To maintain intracellular homeostasis, a highly conserved stress-inducible metabolic protein Sestrin2 is activated to stimulate AMPK-mediated mitochondrial quality control and repress ROS. CSA upregulated Sestrin2/AMPK signaling pathway, leading to enhanced mitochondrial quality control, thereby protects against APAP-induced liver injury.

In conclusion, this study demonstrates that CSA protects against APAP-induced liver injury via enhancing mitochondrial quality control and alleviating oxidative stress, which is possibly associated with Sestrin2-LKB1-AMPK activation (Figure 8). From a clinical standpoint, CSA would be a promising drug for treating APAP-induced liver injury.

## Data Availability

The original contributions presented in the study are included in the article/Supplementary Material, further inquiries can be directed to the corresponding author.
